# Precocious genotypes and homozygous tendency generated by self-pollination in walnut

**DOI:** 10.1186/s12870-018-1549-1

**Published:** 2018-12-04

**Authors:** Lingna Chen, Runquan Dong, Qingguo Ma, Yu Zhang, Shizhong Xu, Delu Ning, Qin Chen, Dong Pei

**Affiliations:** 1 State Key Laboratory of Tree Genetics and Breeding, Key Laboratory of Tree Breeding and Cultivation of the State Forestry and Grassland Administration, Research Institute of Forestry, Chinese Academy of Forestry, Beijing, 100091 China; 2 Research Institute of Resources Insects, Chinese Academy of Forestry, Kunming, 650233 Yunnan China; 3 Yunnan Academy of Forestry, Kunming, 650204 Yunnan China; 4 Dali Forest Resource Management Station, Dali, 671000 Yunnan China

**Keywords:** *Juglans sigillata* Dode, *Juglans regia* L., Self-pollination, Phenotype, Homozygosity, Juvenile period

## Abstract

**Background:**

Observations of precocious (early bearing) genotypes of walnut (*Juglans regia* L.) under natural conditions encouraged us to study the origin and genetic control of these fascinating traits.

**Results:**

In this study, the self-fertility, progeny performance, and simple sequence repeat (SSR) locus variation of iron walnut (*Juglans sigillata* Dode), an ecotype of *J. regia*, were investigated. The average self-pollinated fruit set rate of *J. sigillata* cv. ‘Dapao’ (DP) was 7.0% annually from 1979 to 1982. The average germination rate of self-pollinated seeds was 45.2% during the 4-year period. Most progeny had inbreeding depression. Nine representative self-pollinated progeny (SP_1_–SP_9_), with special or typical traits of DP, were selected. SP_1_–SP_4_ were precocious because they initiated flowers as early as 2 years after germination, compared to the 7–10-yr period that is typical of DP. SP_9_ had not flowered since 1980. Twelve SSR markers were used to analyze the SP and DP. The genome of SP had a tendency toward high levels of homozygosity. The high levels of homozygosity reported in 18 additional precocious walnut genotypes complemented the results of this study.

**Conclusions:**

These results provide evidence of precocious phenotypes and genomes with high levels of homozygosity that might be generated from self-pollinating walnut. This suggests that self-pollination might facilitate the generation of unique homozygous parents for subsequent use in walnut-breeding programs. The results also indicate that more attention should be focused on adequate management of precocious walnut to avoid early depression in the production of nuts.

**Electronic supplementary material:**

The online version of this article (10.1186/s12870-018-1549-1) contains supplementary material, which is available to authorized users.

## Background

Precocity and prolificacy are important in fruit breeding programs, and an understanding of the knowledge is crucial in the assessment of clones that may be released as new cultivars [1–3]. In recent years, dwarf rootstocks and early-producing parents have been used to manage and shorten the juvenile period in many fruit trees, including apple, pear, citrus, olive, cherry, and walnut [2, 4, 5]. Negative correlations between the length of the juvenile period and seedling vigor or tree size have been observed in different woody species [2, 4, 6]. These precocious and dwarf trees have attracted the attention of fruit growers throughout the world because of the potential for increased planting density, higher production and photosynthetic efficiency, effective spraying, and easy of harvesting [7–9].

Walnut (*Juglans regia* L.) is not considered to be a precocious tree [4]. It exhibits vigorous growth, but flowering is rarely initiated within 1–3 yr. of sowing. Early flowering has been preferentially selected in breeding programs to produce precocious cultivars. Rezaee et al. [7] reported that some precocious and dwarf walnut genotypes can be found among the seedlings of some *J. regia* genotypes from western Asia. In China, precocious genotypes have been selected mainly from the Aksu, Hotan and Kashgar regions of Xinjiang, which are situated in the Tarim Basin, in an area characterized by an arid climate, low precipitation, and strong evaporation [10, 11]. However, little is known about the origins of the genotypes. Self-pollination and apomixis occur frequently due to floral variation and limited pollen production, based on the traits of the secondary flowers of precocious *J. regia* [4, 10, 12]. Many plants, including some heterodichogamous taxa, autonomously self-pollinate in natural populations, especially if pollen is limited [13, 14]. Beineke [15] reported that self-pollination could occur when flowering times overlap in black walnut. Self-pollination typically results in broad phenotypic and physiological changes in plants [16]. In fruit crops such as pear (*Pyrus pyrifolia*) [17], almond (*Prunus dulcis*) [18], and some species of the Juglandaceae family, including *Carya illinoensis*, *J. regia*, *J. nigra* and *J. mandshurica* [15, 19], self-pollination causes inbreeding depression and generates dwarfed phenotypes. However, our knowledge of the genetic basis of self-pollination and the phenotype of self-pollinated progeny in the genus *Juglans* L., especially during the juvenile period is limited.

Iron walnut (*Juglans sigillata* Dode) is native to southwestern China and is an ecotype of *J. regia* [20, 21]. It is a deciduous tree species known for its edible nuts and high-quality timber [22]. By the end of 2017, almost 3 million hectares of iron walnut were being harvested in Yunnan Province, and the production value was estimated to be almost 5 billion dollars. Iron walnut is also diploid, monoecious, heterodichogamous (individuals may be protandrous or protogynous), and wind-pollinated. It generally bears fruit approximately 7–10 yr. after sowing. The creation of precocious iron walnut genotype from seedling selection has not been reported in the literature. In this study, a self-pollination experiment was used to determine the self-pollinating capability and to observe the performance of self-pollinated progeny (SP), including the length of the juvenile period and botanical traits, from ‘Dapao’ (DP), an iron walnut cultivar important to Yunnan Province. Simple sequence repeat (SSR) markers were used to infer the genotypic composition of the self-pollinated plants and to understand their genetic traits. The objectives of this study were to provide evidence of new precocious walnut phenotypes, with high levels of homozygosity generated from self-pollination, and to provide guidance for the management of precocious walnut to avoid early depression in nut production.

## Methods

### Plant material

The experiments were conducted in Yangbi County of Dali, Yunnan, China (25°39′N and 100°01′E, 1850 m ASL). DP, a protandrous cultivar of iron walnut and the only genotype within an 800 m radius, was used in controlled pollination experiments from 1979 to 1982. At least 20 trees were selected for bagging each year. Harvested seeds were planted in the experimental walnut orchard of the Yangbi Walnut Research Institute, Yunnan Academy of Forestry, China (25°39′N and 99°59′E). The SP and the parent were grown on the same site. The parent DP and their SP were analyzed using SSR markers.

### Self-pollination of DP and phenotypes of their SP

Female DP flowers were randomly selected and then isolated using a 40 × 60 cm waterproof parchment bag to isolate random pollen grains from other genotypes. Pollen was collected from each genotype and stored in breathable phial at 2–4 °C [23]. All pollen was applied within 48 h to ensure viability. When the stigmas of the bagged female flowers were receptive, pollen was dusted onto them. Pollination was repeated the next day. At 25 days after pollination, bags were removed when the stigmas were completely dry. The number of fruits set was recorded at 8 weeks after anthesis and the self-pollinated fruit setting rate was recorded. The germination rate of the self-pollinated seeds was recorded after the stratification period, and the growth performance attributes of SP, such as the juvenile period, mass growth, and nut traits were continually observed. The un-germinated seeds were separated from the soil for measurement of the empty-nut ratio.

Phenotypic analyses, including the determination of bud, leaf and nut traits of DP and SP were conducted according to the International Union for the Protection of New Varieties of Plants [24] descriptors. During the harvest season, 10 nuts were collected from each tree except SP_9_ for the determination of nut traits (i.e., nut shape, nut size, single nut weight, shell thickness, and kernel percentage). Differences in single nut traits, shell thickness, and kernel percentage among SPs were compared by a one-way ANOVA using SAS (Version 9.2; SAS Institute Inc., Cary, NC, USA).

### SSR analysis of DP and SP

In April 2016, at least six young leaves from the DP and SP plants were collected for extraction of genomic DNA, following the protocol described by Wang et al. [25]. Extracted DNA was quantified spectrophotometrically and diluted to 25 ng/μl before PCR amplification.

Genetic analysis of DP and SP plants was performed using 12 pairs of SSR primers (Table 1) selected for their polymorphisms. Nine primer pairs, prefixed “WJR”, were from bacterial artificial chromosome end sequences of *J. regia* (downloaded from the National Center for Biotechnology Information (NCBI) database: http://www.ncbi.nlm.nih.gov/nucgss/ by querying juglans regia), and three pairs of primers, prefixed “WGA”, were from the enriched (GA/CT)n microsatellite library of *J. nigra* [26]. The SSR reaction protocol was as described by Chen et al. [27]. The amplification reaction was performed in a volume of 20 μL containing 1× polymerase chain reaction (PCR) buffer, 50 ng of genomic DNA, 300 μM dNTPs, 0.4 μM of each primer, 3 mM of MgCl_2_, and 1 unit of Taq DNA polymerase (TaKaRa Biotechnology, Dalian, China). PCR was performed on an ABI GeneAmp®9700 thermal cycler (Applied Biosystems, Foster City, CA, USA) with the following program: an initial 5-min incubation at 94 °C, 35 cycles of 45 s at 94 °C, 45 s at the annealing temperature of 50–55 °C, 45 s at 72 °C, and a final incubation at 72 °C for 5 min. After amplification, 3 μL of each sample were separated in a 6% denaturing polyacrylamide gel with 7 M urea and 1 × TBE (Tris-borate-EDTA) buffer, and visualized by silver staining. In all cases, PCR reactions were performed at least twice to ensure that the absence of bands was not due to a failed reaction.

**Table 1:** Profiles of the 12 pairs of primers used for the SSR analysis of DP and SP

Code	Locus	Repeat motifs	Primer sequences (5′–3′)	GenBank accession no.
1	WJR022	(AAG)n	F: ACGGGACCGGAGTTTACTTT	JM057F12
			R: CATGGCAGGAGAACTGGTTT	
2	WJR033	(TA)n	F: AGGGCTCCACTTGATCAGAA	JM056N06
			R: TCGGCAATCAACCAGATAAA	
3	WJR035	(TA)n	F: AGTGCATGCCTTGTCTCCTT	JM060C06
			R: TGCTCCTTGTCAGTCCACAG	
4	WJR061	(AT)n	F: CAAGACCACAGCACAGCATAA	JM008C04
			R: GGGAGTGCTGGAATCGAATA	
5	WJR087	(TGTC)n	F: CCCCCAATATGTCTGCTTCT	JM012K14
			R: ACCATAGCTGGTTTGGCATC	
6	WJR100	(AT)n	F: CGACGATTCGGTGAAGAAAT	JM031J07
			R: GAAAACCCAGTTTCTGTCGG	
7	WJR265	(AAT)n	F: TGGCTATTGCAAAATCAGGTC	JM021P05
			R: CAAAAGCATGTAGGTCGGGT	
8	WJR294	(CAAAAC)n	F: TTTACCTGCCAACACCAACA	JM017N12
			R: ACAAGGCGAAACAAACTGCT	
9	WJR309	(T)n(TTG)n(TTC)n	F: TTGCAATAATGCGATGAACG	JM006J18
			R: TGACTTTGACCATGGCTTTG	
10	WGA070	(GA)n	F: TGTAATTGGGGAATGTTGCA	–
			R:TGGGAGACACAATGATCGAA	
11	WGA079	(GA)n	F: CACTGTGGCACTGCTCATCT	–
			R: TTCGAGCTCTGGACCACC	
12	WGA089	(TG)n, (GA)n	F:ACCCATCTTTCACGTGTGTG	–
			R:TGCCTAATTAGCAATTTCCA	

The observed heterozygosity (*H*_O_), expected heterozygosity (*H*_E_), *Nei’s* expected heterozygosity (*H*), Shannon’s information index (*I*) of the 12 SSR loci and the allele frequency of DP and SP were calculated using POPGENE version 1.32 [28]. Selfing was assessed by estimating the fixation index (*F*) and the homozygosis (*Hom*) for every SSR locus as *F* = 1 - (*H*_*O*_ / *H*_*E*_) and *Hom* = 1 – *H*_*O*_, respectively, and the statistical significance for *F* values was tested using 1000 permutations of alleles among individuals and Bonferroni correction (95%, *p* = 0.05), following the methods of Bressan et al. [29].

The difference in alleles among SP was evaluated by Student’s *t*-test for independent samples using Excel 2010 (Microsoft Corp., Seattle, WA, USA).

## Results

### Self-pollinating capability of iron walnut ‘Dapao’

DP had relatively stable self-pollinating capability, ranging from 5.4 to 9.3%, with an average of 7.0%, during the 4-yr period (Table 2). A total of 378 self-pollinated seeds were harvested. Among them, embryoless and empty seeds accounted for 31.2 and 23.5% of the harvested seeds, respectively. The remaining 171 (45.2%) seeds germinated after stratifcation treatment. Most of the 171 seeds did not grow properly because of depression. Only 47 of the 171 plants survived and reached reproductive maturity.

**Table 2:** Self-pollinating capability of iron walnut ‘Dapao’

Year	Number of bagged female flowers	Number of fruits set	Fruit setting rate (%)	Number of harvested seeds		
				Number of germinated seeds	Number of embryoless seeds	Number of empty seeds
1979	1722	93	5.4	39	31	23
1980	1688	131	7.7	27	25	34
1981	1786	96	5.4	37	27	17
1982	1379	128	9.3	68	35	15
Total	6575	448	–	171	118	89
Mean	–	–	7.0	–	–	–

### Phenotype variation of SP

The most striking result of self-pollination observed in this study was SP depression, in terms of reduced growth and vitality. There were different levels of phenotypic changes. One trait of the juvenile period should be underlined. Three major categories of seedling were observed among the SP of DP. The length of the juvenile period was shortened from around the 7–10 yr. usually required by iron walnut to a 2-yr period for SP_1_–SP_4_. Also, SP_9_ never flowered. However, many SPs, represented by SP_5_–SP_8_, had similar lengths of juvenile period to their parents. The nine representative seedlings were retained to investigate the variation in botanic traits.

After more than 30 yr. of investigation, the botanic traits of SPs have been found to be basically stable. Growth traits of the nine normal SP plants are shown in Table 3. These plants had different leaflet and nut-shape features. SP_1_–SP_4_ had precocious traits and different stem colors and leaflet numbers compared to DP. SP_5_–SP_8_ exhibited a red rachis color, which was clearly different from the green color in DP and other SPs. SP_6_ had a circular mixed bud, unlike most SPs, which had triangular buds. SP_4_ and SP_6_ appeared to have different dichogamy types. The variation in nut shape was very significant, with three types of nut being apparent: Pao walnut (with 0.1–0.9 mm shell thickness in SP_1_, SP_2_, and SP_6_), Jiamian walnut (with 1.0–1.5 mm shell thickness in SP_3_, SP_4_, and SP_5_), and Tie walnut (with 1.6–2.0 mm and > 2.0 mm shell thickness in SP_7_ and SP_8_, respectively). Shell thickness was significantly negatively related to the kernel percentage, with a very significant difference between SP_7_ and SP_8_ and the six other nut-bearing plants (*p* < 0.01). Among these plants, SP_5_ was most similar to DP in terms of phenotype.

**Table 3:** Phenotypic differences between DP and SP

Trait		DP	SP_1_	SP_2_	SP_3_	SP_4_	SP_5_	SP_6_	SP_7_	SP_8_	SP_9_
Tree	Age of tree (yr)	≈70	36	36	35	34	36	33	36	33	36
	Diameter at breast height (cm)	53.5	10.1	13.8	11.2	14.2	*19.4*	*24.3*	*22.4*	*17.8*	9.3
	Juvenile period (yr)	7	*2*	*2*	*2*	*2*	8	7	8	7	*Never flower*
	Stem color	Brown	Taupe	Taupe	Taupe	Taupe	Taupe	Taupe	*Gray*	Taupe	Brown
	Type of dichogamy	Protandrous	Protandrous	Protandrous	Protandrous	*Protogynous*	Protandrous	*Protogynous*	Protandrous	Protandrous	Protandrous
Bud	Longitudinal section of mixed bud	Triangular	Triangular	Triangular	Triangular	Triangular	Triangular	*Circular*	Triangular	Triangular	Triangular
Leaf	Number of leaflets	7~13	5~9	5~9	5~9	5~9	7~13	7~11	7~13	7~11	9~13
	Leaflet shape	Lanceolate	Elliptic	Elliptic	Elliptic	Oval	Elliptic	Oval	Lanceolate	Lanceolate	Elliptic
	Terminal leaflet	Small	Large	Large	Large	Large	Degradation	Large	Degradation	Degradation	Medium
	Rachis color	Green	Green	Green	Green	Green	*Red*	*Red*	*Red*	*Red*	Green
Nut	Shape in longitudinal section through suture	Oblate	Circular	Circular	Oblate	Ovate	Circular	Oblate	Oblate	Circular	–
	Shape of apical tip	Pointed	Bulge	Pointed	Emarginate	Bulge	Pointed	Bulge	Bulge	Pointed	–
	Shape of apex perpendicular to suture	Truncate	Rounded	Rounded	Emarginate	Truncate	Rounded	Truncate	Truncate	Truncate	–
	Shape of base perpendicular to suture	Rounded	Rounded	Rounded	Truncate	Rounded	Rounded	Truncate	Rounded	Rounded	–
	Nut vertical diameter (mm)	38.70 ± 0.99	35.09 ± 1.05	37.11 ± 1.24	32.29 ± 1.03	34.58 ± 0.88	28.21 ± 1.12	31.78 ± 1.21	31.79 ± 1.14	33.18 ± 1.44	–
	Nut transverse diameter (mm)	38.11 ± 0.89	32.36 ± 1.43	35.40 ± 1.10	36.74 ± 1.05	29.52 ± 0.88	30.80 ± 1.16	37.26 ± 1.20	36.46 ± 1.19	31.81 ± 1.24	–
	Nut side diameter (mm)	31.03 ± 0.66	27.48 ± 1.15	31.17 ± 1.01	28.71 ± 0.91	32.36 ± 0.83	26.79 ± 1.30	32.19 ± 1.21	28.84 ± 1.11	26.73 ± 1.21	–
	Dividing membranes traits	Paper-like	Membranous	Membranous	Leathery	Paper-like	Membranous	Membranous	Bony	Bony	–
	Single nut weight (g)	12.54 ± 0.69	9.08 ± 0.56	11.07 ± 0.75	10.85 ± 1.04	10.86 ± 1.68	8.11 ± 0.85	12.08 ± 1.06	18.32 ± 1.01	13.32 ± 0.86	–
	Shell thickness (mm)	0.96 ± 0.02	0.95 ± 0.03	0.97 ± 0.03	1.17 ± 0.02	1.08 ± 0.02	1.17 ± 0.01	0.98 ± 0.02	1.87 ± 0.05	3.27 ± 0.22	–
	Kernel percentage (%)	55.14 ± 0.99	53.40 ± 0.68	51.12 ± 0.86	48.23 ± 0.96	51.24 ± 0.40	50.57 ± 0.59	58.14 ± 0.85	19.10 ± 1.51	20.07 ± 2.19	–

SP_1_–SP_9_ represented nine self-pollinated plants sown during the 1980–1983 period. Among them, SP_9_ did not flower until 2017. DP and SP exhibited some differences in stem color, number and shape of leaflets, and nut-shape traits. Ten nuts were investigated for nut traits because of the low fruits set. Tree height data are not provided because SP7 and SP9 were truncated in 2010

### SSR analysis of DP and SP

The 12 SSRs produced a total of 24 alleles among DP and SP. Among them, six SSR loci were heterozygous in the mother DP. There were no more than two SSR alleles per SP and DP. No new alleles appeared in SP, suggesting that the genome of SP was derived from DP. No SP displayed the same genotypes as the DP, indicating no apomictic progeny in this sample. The estimated average fixation index for the SP differed significantly from zero (*F* = 0.136, *p* < 0.05). The expected heterozygosity (*H*_E_) values for the 12 SSR loci ranged from 0.100 to 0.526, with an average value of 0.356. The Shannon’s information index (*I*) ranged from 0.199 to 0.693, with an average value of 0.502 (Table 4). The smallest number of alleles (*n* = 13) was observed in SP_3_, and the largest number (*n* = 18) was observed in the DP and SP_5_. The allele frequency ranged from 0.05 to 0.95 (Table 5). Homozygosis (*Hom*) per locus ranged from 0.300 to 0.900. The percentages of homozygous loci in the SP genome were estimated to be 58% (SP_1_), 83% (SP_2_), 92% (SP_3_), 83% (SP_4_), 50% (SP_5_), 58% (SP_6_), 58% (SP_7_), 75% (SP_8_), and 83% (SP_9_), and that in the DP it was 50%. SP had increased homozygosity in the genome. SP_3_ was the highest, while SP_5_ was the lowest and was similar to DP.

**Table 4:** Summary of genetic statistics for 12 SSR loci in DP and SP

Locus	Sample size	*H*_O_	*H*_E_	*H*	*I*	*F*	*Hom*
WJR022	10	0.300	0.268	0.255	0.423	−0.143	0.700
WJR033	10	0.100	0.100	0.095	0.199	0.000	0.900
WJR035	10	0.500	0.479	0.455	0.647	−0.067	0.500
WJR061	10	0.600	0.526	0.500	0.693	−0.053	0.400
WJR087	10	0.300	0.521	0.495	0.688	0.590	0.700
WJR100	10	0.400	0.505	0.480	0.673	0.351	0.600
WJR265	10	0.200	0.190	0.180	0.325	−0.067	0.800
WJR294	10	0.100	0.100	0.095	0.199	0.000	0.900
WJR309	10	0.100	0.100	0.095	0.199	0.000	0.900
WGA070	10	0.500	0.479	0.455	0.647	0.059	0.500
WGA079	10	0.700	0.479	0.455	0.647	−0.455	0.300
WGA089	10	0.300	0.521	0.495	0.688	0.590	0.700
Mean	10	0.342	0.356	0.338	0.502	0.136	0.658
St.dev.		0.202	0.187	0.177	0.216

**Table 5:** Allele frequency of DP and SP

Allele\Locus	WJR022	WJR033	WJR035	WJR061	WJR087	WJR100
Allele A	0.15	0.95	0.35	0.50	0.55	0.40
Allele B	0.85	0.05	0.65	0.50	0.45	0.60
Allele\Locus	WJR265	WJR294	WJR309	WGA070	WGA079	WGA089
Allele A	0.90	0.95	0.05	0.35	0.35	0.45
Allele B	0.10	0.05	0.95	0.65	0.65	0.55

Student’s *t*-test was used to evaluate allele differences in the 12 SSR loci between the short (SP_1_–SP_4_) and long (SP_5_–SP_8_) juvenile periods. A *P* value of 0.080 (*t* = − 2.102, df = 6) was obtained, indicating a large difference between the two groups.

Twelve SSR primers were used to analyze genetic diversity, allele frequency, and the number of homozygous loci in 36 additional *J. regia* genotypes (Additional file 1), including 18 that initiated flowering within 1–3 yr. of sowing and 18 that initiated flowering more than 4 yr. after sowing. The expected heterozygosity (*H*_E_) and Shannon’s information index (*I*) of the 12 SSR loci had average values of 0.623 and 1.129, respectively, indicating a high degree of polymorphism in the SSR loci (Additional file 2). The estimated average fixation index was also differed significantly from zero (*F* = 0.333, *p* < 0.05). SSR genotyping revealed a significant difference in the level of homozygosity among groups (*t* = 6.204, df = 38, *P* = 0). The percentage of homozygous loci was larger in the precocious group (64.90%) than in the long juvenile period group (47.85%; Additional file 3). The percentages of homozygous loci exceeded 70% in four cultivars: ‘Liaoning 2’, ‘Lvbo’, ‘Zha 71’, and ‘Chandler’.

## Discussion

### Self-pollination and self-compatibility of walnut

Self-compatibility is common in heterodichogamous taxa, including *Juglans* and *Acer* [13, 30]. In the present study, an average self-pollinated fruit setting rate of 7.0% was recorded for ‘Dapao’ during the 4-yr experiment, suggesting that the species might be self-compatible, like other species of *Juglans* [13, 31] or there is individual variation for self-incompatibility and some trees might set seed from self-fertilization, where other not. Whatever the case, the selfing capacity has been applied in breeding programs for self-pollinated fruit crops, such as cherry [32]. Conveniently, self-pollination can occur in natural populations of walnuts, especially if pollen dispersal distance is limited [14, 19, 33]. In southern Xinjiang regions, such as Kashgar and Hotan, frequent sandstorms and severe droughts might seriously interfere with the cross-pollination of walnut in April and May, which corresponds to the blooming stage. As a result, the proportion of selfed or inbred progeny from climate-stress trees in the seed gene pool might be dramatically increase in the region, which was also noted in a study of seed productive responses to drought stress in *Pinus sylvestris* [34]. Self-pollination and apomixis occur frequently due to floral variation and limited pollen among the secondary flowers of the precocious *J. regia* [4, 10, 12], although the study did not provide evidence of the generation of the precocious genotype from apomixis. The special climate of southern Xinjiang might enable the formation of precocious walnuts [10]. The self-pollinating capacity could compensate for the negative effects of limited pollen or provide actual reproductive assurance in this ecological contexts [35, 36].

However, in other woody plants, such as *Ceiba pentandra* and *Davidia involucrata*, self-pollination or inbreeding can result in the expression of lethal or deleterious alleles that cause the production of aborted and un-germinated seeds [37, 38]. In this study, the 45.2% germination rate of self-pollinated seeds was lower than that of black walnut (72.7%), as reported by Beineke [15]. Survival of only an estimated 25% of the self-pollinated plants was due to poor fitness, which was attributed to inbreeding depression. The self-pollinating capacity remains vital to the evolution of walnut; self-pollination can be tolerated by some plant species because many mildly deleterious alleles that could contribute to inbreeding depression were previously purged through natural selection [16, 39, 40]. Bressan et al. [29] reported that not all individuals produced from selfing were eliminated by inbreeding depression after germination (post-zygotic control). This is also an important trait in autogamous species [41]. The low vigor and high homozygous genotypes caused by self-pollination could provide the genetic material for tree size control and cross breeding [8, 41].

### Phenotypes and genotypes of SP

Phenotypic variation in SP was observed in this study. More specifically, several precocious genotypes, flowering after 2 yr., were induced by self-pollination. The growth of those plants was substantially reduced compared to plants that had a juvenile period similar to their parent. The shell thickness of their nuts also varied markedly from 0.9–1.0 to 3.0–3.5 mm. There are few literatures reporting the effects of self-pollination on the juvenile period and fruit traits of plants. Lahav et al. [42] concluded that there were no differences in the length of the juvenile period among *Persea americana* progeny that originated from self-pollination and outcrossing. However, self-pollinated plants with low vigor and growth have been observed in some perennial plants, including *J. regia* and *J. nigra* [15]. A significant relationship between growth vigor and precocity levels has been reported in fruit trees, including pecan, olive, citrus, and walnut [2, 4, 6, 43]. The slow growth and short stature (4.5 to 5.5 m) of the four SP were consistent with previous reports. The differences in self-pollination effects among phenotypes and growth stages may reflect differences in either the number or type of deleterious mutations or the loss of the heterozygote advantage [38, 39].

SSRs are ideal markers for characterizing relationships and analysis of variation between walnut individuals [44–46]. The genotype analysis based on SSR marker data revealed an increased homozygosity in SP compared with DP. The degree and type of homozygosity differed among the SP genomes, which might reflect differences in the accumulation of mutations and adaptability [40, 41]. An earlier study on *Persea americana* investigated whether self-pollinations could be deployed to shorten the juvenile period, and indicated no significant effects of the homozygosity level on the length of the juvenile period [41]. Results from a study on *Cocos nucifera* suggested a relationship between a short juvenile period and higher homozygosity [47]. In our supplementary study, SSR genotyping of several precocious walnut cultivars, including ‘Liaoning 2’, ‘Lvbo’, and ‘Zha 71’, which are cultivated widely in China, resulted in a high proportion of homozygous loci (> 70% of SSR loci analyzed; Additional file 3). Although we cannot rule out the possibility that the differences in the juvenile period among SPs in our experiment were caused by increased homozygosity, our results were more supportive of a short juvenile period in connection with higher homozygosity in the genome.

For SPs, the estimated average fixation index was 0.136, which was significantly below the minimum expected value of 0.5 for selfing [29]. This might be related to the fact that the present analysis was based in germinated seeds, probably subject of early inbreeding depression, killing some inbreed genotype combinations and natural selection favouring some inbreed genotypes, although the small family sample size very probably have also affected the inheritance of maternal alleles. For heterozygous loci in the mother, the expected *H*_E_ within families would be more close to 0.5. However, more important, in general homozygous genotypes were favored (> 0.5), resulting in higher homozygous than heterozygous genotypes, with exception of loci WJR061 and WGA079, there the opposite was detected. Therefore, the selection favouring homozygous genotypes might be the main cause of the differences between the observed (0.136) than expected F values (0.5). The similar result is also observed among natural walnut population in Iran, and there is inbreeding among the sampled populations [44, 46, 48].

In summary, the juvenile period was influenced by the combined effects of many recessive variants dispersed across the genome. This might also be true for other tree traits, and represents a new means of understanding the influence of genetic variation on tree performance.

### Implications of self-pollination for walnut breeding

In the present study, iron walnut self-pollination contributed to a shortened juvenile phase in SP. This is the first evidence that self-pollination produces precocious genotypes in iron walnut. Thus, it seems that self-pollination can benefit traditional plant breeding programs [39, 49]. However, the precocity due to increased homozygosity and fitness reduction caused by self-pollination suggest that this is a depressed trait. Precocity has been widely used in breeding programs in China, and seems to be ideal for use in northern China [11, 12]. These facts also suggest that more attention should be focused on strengthening management of water and fertilizers, controlling a reasonable number of fruit-bearing flowers and selecting rootstock with strong growth for precocious walnut to avoid early depression.

On the other hand, self-pollination can cause increased homozygosity of individuals, which can be tested for their usefulness as new cultivars and/or enhance the fitness of their offspring when intercrossed with other germplasms [40]. Thus, plants possessing a short juvenile period and partially homozygous genome induced by self-pollination would create unique homozygous parents and be an excellent germplasm resource for iron walnut breeding.

In summary, these results provide evidence for the origin and heritability of precocious walnut and signs for recognition and utilization of self-pollination in walnuts. Also, the results suggest a new method of generating genetic variation in walnut breeding.

## Additional files

Additional file 1: Detailed information of 18 walnut genotypes with short juvenile period and 18 walnut genotypes with long juvenile period. (DOC 42 kb)

Additional file 2: Summary of genetic statistics for 12 SSR loci in the 36 genotypes analyzed. (DOC 44 kb)

Additional file 3: Percentage of homozygous loci in the 36 genotypes analyzed. (DOC 38 kb)

## References

[1] Karelia V, Jesús A, Vives MC, Pablo A, Pina JA, Pedro M, Luis N, Guerri J (2016). Precocious flowering of juvenile citrus induced by a viral vector based on Citrus leaf blotch virus: a new tool for genetics and breeding. Plant Biotechnol J.

[2] Rallo P, Jiménez R, Ordovás J, Suárez MP (2008). Possible early selection of short juvenile period olive plants based on seedling traits. Aust J Agric Res.

[3] Moral J, Díez CM, León L, De la Rosa R, Santos-Antunes F, Barranco D, Rallo L (2013). Female genitor effect on the juvenile period of olive seedlings. Sci Hortic.

[4] Vahdati K, Mohseniazar M (2016). Early bearing genotypes of walnut: a suitable material for breeding and high density orchards. Acta Hortic.

[5] Continella A, Pannitteri C, Malfa SL, Legua P, Distefano G, Nicolosi E, Gentile A (2018). Influence of different rootstocks on yield precocity and fruit quality of ‘Tarocco Scirè’ pigmented sweet orange. Sci Hortic.

[6] Thompson TE, Grauke LJ (2003). Pecan tree growth and precocity. J Am Soc Hortic Sci.

[7] Rezaee R, Vahdati K, Valizadeh M (2009). Variability of seedling vigour in persian walnut as influenced by the vigour and bearing habit of the mother tree. J Hortic Sci Biotechnol.

[8] Vahdati K, Mirmasoumi M, Rezaee R (2009). Rooting and multiplication ability of Persian walnut as influenced by motherstock vigor and precocity. Acta Hortic.

[9] Foster TM, Mcatee PA, Waite CN, Boldingh HL, Mcghie TK (2017). Apple dwarfing rootstocks exhibit an imbalance in carbohydrate allocation and reduced cell growth and metabolism. Hortic Res.

[10] Li M, Liu Y, Zhao Y, Sun M, Zhang X, Yang K, Guo Q (2009). Studies on floral variation in walnut (*Juglans regia* L.). Acta Hortic Sinica.

[11] Wang GA, Zhang Q, Huang MM, Yakup A (2013). The breeding of six Xinjiang dwarf walnut cultivars. Acta Hortic.

[12] Bernard A, Lheureux F, Dirlewanger E (2018). Walnut: past and future of genetic improvement. Tree Genet Genomes.

[13] Kimura MK, Goto S, Suyama Y, Matsui M, Woeste K, Seiwa K (2012). Morph-specific mating patterns in a low-density population of a heterodichogamous tree, *Juglans ailantifolia*. Plant Ecol.

[14] Peng DL, Ou XK, Xu B, Zhang ZQ, Niu Y, Li ZM, Sun H (2014). Plant sexual systems correlated with morphological traits: reflecting reproductive strategies of alpine plants. J Syst Evol.

[15] Beineke WF (1987). The effects of inbreeding in black walnut. Proceedings of the Indiana Academy of Science.

[16] Zhang M, Zhou L, Bawa R, Suren H, Holliday JA (2016). Recombination rate variation, hitchhiking, and demographic history shape deleterious load in poplar. Mol Biol Evol.

[17] Sato A, Sawamura Y, Takada N, Hirabayashi T (2008). Relationship between inbreeding coefficients and plant height of 1-year-old seedlings in crosses among Japanese pear (*Pyrus pyrifolia* Nakai) cultivars/selections. Sci Hortic.

[18] Martínez-García PJ, Dicenta F, Ortega E (2011). Anomalous embryo sac development and fruit abortion caused by inbreeding depression in almond (*Prunus dulcis*). Sci Hortic.

[19] Bai WN, Zeng YF, Zhang DY (2007). Mating patterns and pollen dispersal in a heterodichogamous tree, *Juglans mandshurica* (Juglandaceae). New Phytol.

[20] Wang H, Pan G, Ma QG, Zhang JP, Pei D (2015). The genetic diversity and introgression of *Juglans regia* and *Juglans sigillata* in Tibet as revealed by SSR markers. Tree Genet Genomes.

[21] Zhao Peng, Zhou Hui-Juan, Potter Daniel, Hu Yi-Heng, Feng Xiao-Jia, Dang Meng, Feng Li, Zulfiqar Saman, Liu Wen-Zhe, Zhao Gui-Fang, Woeste Keith (2018). Population genetics, phylogenomics and hybrid speciation of Juglans in China determined from whole chloroplast genomes, transcriptomes, and genotyping-by-sequencing (GBS). Molecular Phylogenetics and Evolution.

[22] Pei D, Lu XZ (2011). Walnut germplasm resources in China.

[23] Liu WS (1963). Preliminary report on experiments on germination of walnut pollen. Sci Silvae Sin.

[24] TG/125/6. Guidelines for the conduct of tests for distinctness, uniformity and stability of *Juglans regia* L. (Walnut). International Union For The Protection Of New Varieties Of Plants (UPOV); 1999.

[25] Wang H, Pei D, Gu RS, Wang BQ (2008). Genetic diversity and structure of walnut populations in central and southwestern China revealed by microsatellite markers. J Am Soc Hortic Sci.

[26] Woeste K, Burns R, Rhodes O, Michler C (2002). Thirty polymorphic nuclear microsatellite loci from black walnut. J Hered.

[27] Chen LN, Ma QG, Chen YK, Wang BQ, Pei D (2014). Identification of major walnut cultivars grown in China based on nut phenotypes and SSR markers. Sci Hortic.

[28] Yeh FC, Yang R, Boyle T (1999). POPGENE Version 1.32 Microsoft Windows-based freeware for populations genetic analysis.

[29] Bressan EA, Sebbenn AM, Ferreira RR, Lee TSG, Figueira A (2013). J*atropha curcas* L. (Euphorbiaceae) exhibits a mixed mating system, high correlated mating and apomixis. Tree Genet Genomes.

[30] Kikuchi S, Shibata M, Tanaka H (2015). Effects of forest fragmentation on the mating system of a cool-temperate heterodichogamous tree *Acer mono*. Glob Ecol Conserv.

[31] Victory ER, Glaubitz JC, Rhodes JOE, Woeste KE (2006). Genetic homogeneity in *Juglans nigra* (Juglandaceae) at nuclear microsatellites. Am J Bot.

[32] Schuster M, Grafe C, Wolfram B, Schmidt H (2014). Cultivars resulting from cherry breeding in Germany. Erwerbs-obstbau.

[33] Mohsenipoor S, Vahdati K, Amiri R, Mozaffari M (2010). Study of the genetic structure and gene flow in Persian walnut (*Juglans regia* L.) using SSR markers. Acta Hortic.

[34] Kuznetsova NF (2015). Development of specific and nonspecific reponses to stress in *Pinus sylvestris* L. at population level in a gradient of drought years. Russ J Ecol.

[35] Delmas CE, Cheptou PO, Escaravage N, Pornon A (2014). High lifetime inbreeding depression counteracts the reproductive assurance benefit of selfing in a mass-flowering shrub. BMC Evol Biol.

[36] Pellegrino G, Bellusci F, Palermo AM (2015). Effects of population structure on pollen flow, clonality rates and reproductive success in fragmented *Serapias lingua* populations. BMC Plant Biol.

[37] Lobo JA, Jiménez D, Solíshernández W, Fuchs EJ (2015). Lack of early inbreeding depression and distribution of selfing rates in the neotropical emergent tree *Ceiba pentandra*: assessment from several reproductive events. Am J Bot.

[38] Meng L, Dong X, Peng J, Wen X, Rui R, Liu J, Gao F, Liu Z (2016). *De novo* transcriptome sequencing and gene expression analysis reveal potential mechanisms of seed abortion in dove tree (*Davidia involucrate* Baill.). BMC Plant Biol.

[39] Charlesworth D, Willis JH (2009). The genetics of inbreeding depression. Nat Rev Genet.

[40] González AM, De Ron AM, Lores M, Santalla M (2014). Effect of the inbreeding depression in progeny fitness of runner bean (*Phaseolus coccineus* L.) and it is implications for breeding. Euphytica.

[41] Debnath S, Guha S, Sharangi A, Datta S (2015). Breeding methods for quality improvement in horticultural crops. Value addition of horticultural crops: recent trends and future directions.

[42] Lahav E, Lavi U, Jain SM, Priyadarshan PM (2009). Avocado genetics and breeding. Breeding plantation tree crops: tropical species.

[43] Mitani N, Matsumoto R, Yoshioka T, Kuniga T (2008). Citrus hybrid seedlings reduce initial time to flower when grafted onto shiikuwasha rootstock. Sci Hortic.

[44] Karimi R, Ershadi A, Vahdati K, Woeste K (2010). Molecular characterization of Persian walnut populations in Iran with microsatellite markers. Hortscience.

[45] Francesca Pop I, Cristina Vicol A, Botu M, Andrei Raica P, Vahdati K, Pamfil D (2013). Relationships of walnut cultivars in a germplasm collection: comparative analysis of phenotypic and molecular data. Sci Hortic.

[46] Vahdati Kourosh, Mohseni Pourtaklu Somayeh, Karimi Rouhollah, Barzehkar Rouhollah, Amiri Reza, Mozaffari Mohammad, Woeste Keith (2014). Genetic diversity and gene flow of some Persian walnut populations in southeast of Iran revealed by SSR markers. Plant Systematics and Evolution.

[47] Batugal P, Bourdeix R. Conventional coconut breeding. In: Batugal PA, Ramanatha Rao V, Oliver J, editors. Coconut genetic resources. Rome: IPGRI; 2005.

[48] Karimi R, Ershadi A, Vahdati K, Ehtesham Nia A, Sharifani M, Rasouli M, Ebrahimi A (2014). Morphological and molecular evaluation of Persian walnut populations in northern and western regions of Iran. J Nuts.

[49] Ramírez N, Nassar JM (2017). Breeding systems in angiosperms: novel inferences from a new analytical approach. Plant Syst Evol.

