# Supplementary material for: Precocious genotypes and homozygous tendency generated by self-pollination in walnut
Source: BMC Plant Biol. 2018 Dec 4;18:323. doi: 10.1186/s12870-018-1549-1 (PMC6278120; doi:10.1186/s12870-018-1549-1)
Supplement: Supplementary file 1 — Detailed information of 18 walnut genotypes with short juvenile period and 18 walnut genotypes with long juvenile period. (DOC 42 kb) [file 12870_2018_1549_MOESM1_ESM.doc]

Detailed information of 18 walnut genotypes with short juvenile period and 18 walnut genotypes with long juvenile period.

| Short juvenile period genotype name | Origin |
| --- | --- |
| Liaoning 1 | Selection of ‘Changlidabaopi’ × selection of ‘Xinjiang paper-shell walnut’ |
| Liaoning 2 | Selection of ‘Changlidabaopi’ × selection of ‘Xinjiang paper-shell walnut’ |
| Liaoning 3 | Selection of ‘Changlidabaopi’ × selection of ‘Xinjiang paper-shell walnut’ |
| Liaoning 4 | ‘Damahetao’ × selection of ‘Xinjiang paper-shell walnut’ |
| Luguang | ‘Kakazi’ × ‘Shangsong 6’ |
| Beijing 861 | Selected from progenies of walnuts from Xinjiang (Xinjiang walnuts) |
| Lvbo | Selected from progenies of Xinjiang walnuts |
| Zha 343 | Selected from progenies of Xinjiang walnuts |
| Baofeng | Selected from progenies of Xinjiang walnuts |
| Zha 210 | Selected from progenies of Xinjiang walnuts |
| Zha 200 | Selected from progenies of Xinjiang walnuts |
| Zha 71 | Selected from progenies of Xinjiang walnuts |
| Xinjiang 1 | Selected from progenies of Xinjiang walnuts |
| Xifu 1 | Selected from progenies of ‘Genianhetao’ |
| Chico | Introduced from United States |
| Amigo | Introduced from United States |
| Chandler | Introduced from United States |
| Pedro | Introduced from United States |
| Long juvenile period genotype name | Origin |
| Jingxiang 1 | Selected from walnut population in Beijing |
| Huashan 5 | Selected from walnut population in Beijing |
| Xisiyu 1 | Selected from walnut population in Beijing |
| Jingxiang 2 | Selected from walnut population in Beijing |
| Jingxiang 3 | Selected from walnut population in Beijing |
| Beijing 1 | Selected from walnut population in Beijing |
| Beijing 2 | Selected from walnut population in Beijing |
| Beijing 3 | Selected from walnut population in Beijing |
| Beijing 4 | Selected from walnut population in Beijing |
| Shangsong 6 | Selected from progenies of Xinjiang walnuts |
| Shangsong 9 | Selected from progenies of Xinjiang walnuts |
| Walnut king | Selected from progenies of Xinjiang walnuts |
| Xinjiang 2 | Selected from progenies of Xinjiang walnuts |
| Xinjiang 3 | Selected from progenies of Xinjiang walnuts |
| Xinjiang 4 | Selected from progenies of Xinjiang walnuts |
| Yunnan 1 | Selected from walnut population in Yunnan |
| Yunnan 2 | Selected from walnut population in Yunnan |
| Franquette | Introduced from United States |
