# Supplementary material for: Precocious genotypes and homozygous tendency generated by self-pollination in walnut
Source: BMC Plant Biol. 2018 Dec 4;18:323. doi: 10.1186/s12870-018-1549-1 (PMC6278120; doi:10.1186/s12870-018-1549-1)
Supplement: Supplementary file 2 — Summary of genetic statistics for 12 SSR loci in the 36 genotypes analyzed. (DOC 44 kb) [file 12870_2018_1549_MOESM2_ESM.doc]

Summary of genetic statistics for 12 SSR loci in the 36 genotypes analyzed.

| Locus | Sample Size | *H*_O_ | *H*_E_ | *H* | *I* | *F* | *Hom* |
| --- | --- | --- | --- | --- | --- | --- | --- |
| WJR022 | 36 | 0.326 | 0.674 | 0.658 | 1.186 | 0.517 | 0.674 |
| WJR033 | 36 | 0.489 | 0.512 | 0.499 | 0.927 | 0.045 | 0.512 |
| WJR035 | 36 | 0.558 | 0.442 | 0.431 | 0.797 | -0.261 | 0.442 |
| WJR061 | 36 | 0.223 | 0.777 | 0.758 | 1.650 | 0.713 | 0.777 |
| WJR087 | 36 | 0.451 | 0.549 | 0.535 | 0.845 | 0.178 | 0.549 |
| WJR100 | 36 | 0.274 | 0.726 | 0.708 | 1.357 | 0.622 | 0.726 |
| WJR265 | 36 | 0.205 | 0.795 | 0.775 | 1.643 | 0.742 | 0.795 |
| WJR294 | 36 | 0.569 | 0.431 | 0.420 | 0.611 | -0.321 | 0.431 |
| WJR309 | 36 | 0.369 | 0.631 | 0.615 | 1.094 | 0.415 | 0.631 |
| WGA070 | 36 | 0.286 | 0.714 | 0.696 | 1.276 | 0.600 | 0.714 |
| WGA079 | 36 | 0.358 | 0.642 | 0.626 | 1.224 | 0.443 | 0.642 |
| WGA089 | 36 | 0.412 | 0.589 | 0.574 | 0.936 | 0.301 | 0.589 |
| Mean | 36 | 0.377 | 0.623 | 0.608 | 1.129 | 0.333 | 0.623 |
| St. | Dev | 0.122 | 0.122 | 0.119 | 0.325 |  |  |
