# Supplementary material for: Precocious genotypes and homozygous tendency generated by self-pollination in walnut
Source: BMC Plant Biol. 2018 Dec 4;18:323. doi: 10.1186/s12870-018-1549-1 (PMC6278120; doi:10.1186/s12870-018-1549-1)
Supplement: Supplementary file 3 — Percentage of homozygous loci in the 36 genotypes analyzed. (DOC 38 kb) [file 12870_2018_1549_MOESM3_ESM.doc]

Percentage of homozygous loci in the 36 genotypes analyzed.

| Short juvenile period genotypes | | Long juvenile period genotypes | |
| --- | --- | --- | --- |
| Genotype name | Percentage of homozygous loci (%) | Genotype name | Percentage of homozygous loci (%) |
| Liaoning 1 | 66.67 | Jingxiang 1 | 50.00 |
| Liaoning 2 | 75.00 | Huashan 5 | 41.67 |
| Liaoning 3 | 63.64 | Xisiyu 1 | 50.00 |
| Liaoning 4 | 58.33 | Jingxiang 2 | 41.67 |
| Luguang | 54.55 | Jingxiang 3 | 45.45 |
| Beijing 861 | 54.55 | Beijing 1 | 50.00 |
| Lvbo | 72.73 | Beijing 2 | 41.67 |
| Zha 343 | 58.33 | Beijing 3 | 50.00 |
| Baofeng | 58.33 | Beijing 4 | 58.33 |
| Zha 210 | 66.67 | Shangsong 6 | 41.67 |
| Zha 200 | 66.67 | Shangsong 9 | 58.33 |
| Zha 71 | 72.73 | Walnut king | 25.00 |
| Xinjiang 1 | 66.67 | Xinjiang 2 | 58.33 |
| Xifu 1 | 66.67 | Xinjiang 3 | 45.45 |
| Chico | 58.33 | Xinjiang 4 | 36.36 |
| Amigo | 58.33 | Yunnan 1 | 45.45 |
| Chandler | 83.33 | Yunnan 2 | 58.33 |
| Pedro | 66.67 | Franquette | 63.64 |
| Mean | 64.90 | Mean | 47.85 |
